# Manipulating electronic structure of graphene for producing ferromagnetic graphene particles by Leidenfrost effect-based method

**DOI:** 10.1038/s41598-020-63478-7

**Published:** 2020-04-23

**Authors:** Mahsa Alimohammadian, Beheshteh Sohrabi

**Affiliations:** Department of Chemistry, Surface Chemistry Research Laboratory, Iran University of Science and Technology, 16846-13114 Tehran, Iran

**Keywords:** Electronic properties and devices, Synthesis of graphene

## Abstract

First isolation of graphene, as a great achievement, opens a new horizon in a broad range of science. Graphene is one of the most promising materials for spintronic fields whose application is limited due to its weak magnetic property. Despite many experimental and theoretical efforts for obtaining ferromagnetic graphene, still, a high degree of magnetization is an unsolved challenge. Even, in most observations, graphene magnetization is reported at extremely low temperatures rather than room temperature. In principle, the magnetic property of graphene is created by manipulation of its electronic structure. Removing or adding bonds of graphene such as creating vacancy defects, doping, adatom, edges, and functionalization can change the electronic structure and the external perturbation, such as external magnetic field, temperature, and strain can either. Recently, single and few-layer graphene have been investigated in the presence of these perturbations, and also the electronic changes have been determined by Raman spectroscopy. Here, we successfully could develop a simple and novel Leidenfrost effect-based method for graphene magnetization at room temperature with the external perturbations which apply simultaneously in the graphene flakes inside the Leidenfrost droplets. Macroscale ferromagnetic graphene particles are produced by this method. Briefly, the graphene is obtained by the liquid-phase exfoliation method in the ethanol solution media and also evaporates on the hot surface as a Leidenfrost droplet in the magnetic fields. Then, the floated graphene flakes circulate inside the droplets. Due to the strain and temperature inside the droplets and external magnetic field (the magnet in heater-stirrer), the electronic structure of graphene is instantly changed. The changes are extremely rapid that the graphene flakes behave as a charged particle and also produce an internal magnetic field during their circulation. The internal magnetic field is measured by sensors. As the main accomplishment of this study, we could develop a simple method for inducing magnetism obtained 0.4 emu/g in the graphene, as magnetization saturation at room temperature, which is higher than the reported values. Another achievement of this work is the detection of the Leidenfrost droplets magnetic field, as an internal one which has obtained for the first time. To investigate magnetic graphene particles, the magnetization process, and the electronic structure of the vibrating sample magnetometer (VSM), magnetic field sensor, and Raman spectroscopy are used, respectively.

## Introduction

Graphene as an ideal material have unique physical properties and extensive usages. The isolation of single-layer graphene in 2004 was a starting point for exploring its structure and properties^1^. For example, recent studies indicate that the monolayer graphene is optically transparent and can absorb ~2.3% of the visible light^2^, and also it is wetting-transparent to substrates such as copper, gold or silicon^3^. In both cases, transparency is reduced by increasing the number of graphene layers^2,3^. Because of the high flexibility of graphene, the folded, wrinkled, and crumpled graphene can be easily created. Consequently, in these structures, the roughness and specific surface area are raised^4–9^. Moreover, the results of measuring some properties extremely depend on the measurement conditions, such as temperature, the number of layers, and fabrication method^1,10–20^. Suspended and supported fabrication strategies are two types of developing methods for obtaining the free-standing form of graphene. The suspended form can remarkably enhance its electrical and thermal properties because it can effectively eliminate substrate phonon interaction. Generally, the intrinsic properties of graphene are detected in the suspended form without inconvenience of the substrate. For comparison, the electron mobility of suspended graphene is 200,000^12^ cm^2^/Vs and for unsuspended graphene supported by Si/SiO_2_ substrate is 10,000 cm^2^/Vs^1^. Also, the thermal conductivity for suspended monolayer graphene over a trench in Si/SiO_2_ substrate is 5300 W/mK^10^ (at room temperature) and is 600 W/mK^20^ for the supported graphene (near room temperature).

Among all of these properties, the ferromagnetic property of graphene and graphite is extremely low. The negligible amount of their magnetization relate to defects and impurities^21–24^. Recently, many methods have been introduced to change the electronic structure and induce magnetization in graphene. For example, unsaturated dangling bond in vacancy defect is responsible for magnetic moment, which is theoretically estimated about 1.12–1.53 μ_B_ per atom^25,26^. Vacancy defect in graphene and graphite structure is generated by ion (H^+^, He^+^, C^4+^, Ar^+^) irradiation as well as reduction of graphene oxide^24,27–31^. In addition, adatom such as hydrogen and fluorine can create magnetism in graphene by forming the local magnetic moments^26,27,32–37^. Recently, low magnetization has been reported for fluorinated graphene (0.2 emu/g at 1.8 K)^27^, hydrogenated epitaxial one (<30 * 10^−7^ emu/g)^32^, and defective one (0.02 emu/g at 2 K)^27^. Moreover, the zigzag edges in the specific structure of graphene such as graphene nanoribbons have spin-polarized states at the edges which induce magnetism^34,38,39^. Frequently, the graphene oxide is used in the experimental study. The magnetization of ferromagnetic nitrogen-doped graphene created through reduction of graphene oxide in ammonia is 1.66 emu/g (at 2 K, no ferromagnetic at room temperature)^40^, and in nitrogen plasma^41^ is 0.01 emu/g (at room temperature). Using annealing, graphene oxide is reduced in the presence of hydrazine which a magnetization about 0.02 emu/g (at room temperature) was reported by Y. Wang et.al in 2009^28^. The fluorination of the reduced graphene oxide modifies the magnetization up to 2 emu/g at 2 K^33^. In general, there are limitations in the use of graphene oxide and defective graphene. When graphene is oxidized and reduced, its electrical conductivity is disrupted, and when the defect is created in graphene, in high density of defects, the structural stability become loosed and fragile^27,42^. J. Tucek et.al doped graphene by Sulfur and reported its ferromagnetic properties below 62 K (5.5 emu/g) while they observed no ferromagnetic properties at room temperature^43^. Ultimately, modification of graphene magnetization is followed up by functionalizing with nitrophenyl^44^. But most of the studies do not achieve suitable ferromagnetic. In general, ferromagnetic graphene is a matter of interest in the spintronic field^45^. Because the electron structure differs only in the vicinity of the defects, and there are also limitations in the production of these defects, these methods have a low potential for improvement.

In this method, using floated graphene in ethanol droplet during the Leidenfrost effect-based method under the magnetic field at room temperature, the ferromagnetic graphene particles (FGPs) are produced that the magnetization saturation degree is stunningly high in compare to the recent reports. The graphene suspensions are manipulated by Leidenfrost effect. What is important is that the graphene structure is not disrupted by this method.

When a liquid droplet contact to the hot surface, which the surface temperature is upper than the liquid boiling point, an insulating vapor layer is formed that protects the droplet from boiling rapidly. The droplet floats above its own vapor and become shrinkage during evaporation until the liquid become totally dry. This phenomenon was firstly considered by J.G Leidenfrost in 1756 and known as the Leidenfrost effect^46^. Recently, the Leidenfrost droplet has been used to fabricate nanostructures^47^, create photonic microgranules^48^, accelerate the chemical reaction^49^, and used as a chemical reactor^50,51^, additionally, many models have been presented for the Leidenfrost droplet^52–54^. Despite many studies, dynamics such as vibration, rotation, and internal circulation in the droplet still have not been well understood.

## Experimental

### Dispersion of graphene

To disperse graphene, the liquid-phase exfoliation method in ethanol/water solution is used^55^. Adjusting surface tension of solution in the region of 40–50 mN/m is determined as a main parameter for achieving high yield of graphene dispersion^56^. To adjust surface tension, various liquids and surfactants are used^55–60^. In our previous work, the graphene was dispersed in two different surfactants and also their mixture^59^. In this study, in order to reach the surface tension ~45 mN/m, the ratio of 20:80 ethanol/water was used (Supplementary Fig. S1). The graphite (5 gr/lit) was sonicated in 20:80 ethanol/water at high power (400 w) for 30 min by tip ultrasonic. To eliminate larger flakes, the suspensions were centrifuged at 500, 1000, 2000, 3000, 4000, 5000 rpm for 10 min, which is according to the Beer-Lambert equation, the graphene concentration was determined 6.9, 6.1, 4.1, 2.9, 2.1, and 1.8 mM, respectively (Supplementary Fig. S2). After the centrifugation process, to use graphene dispersion in the Leidenfrost effect-based method, the supernatant was decanted and stored in container. In graphene suspensions, graphene has been produced without functionalization with the minimum yield of about 0.66% (for 5000 rpm). These suspensions, as a precursor for Leidenfrost effect-based method, do not create residual chemicals on the FGPs.

### Leidenfrost effect-based method

**Figure 1:**
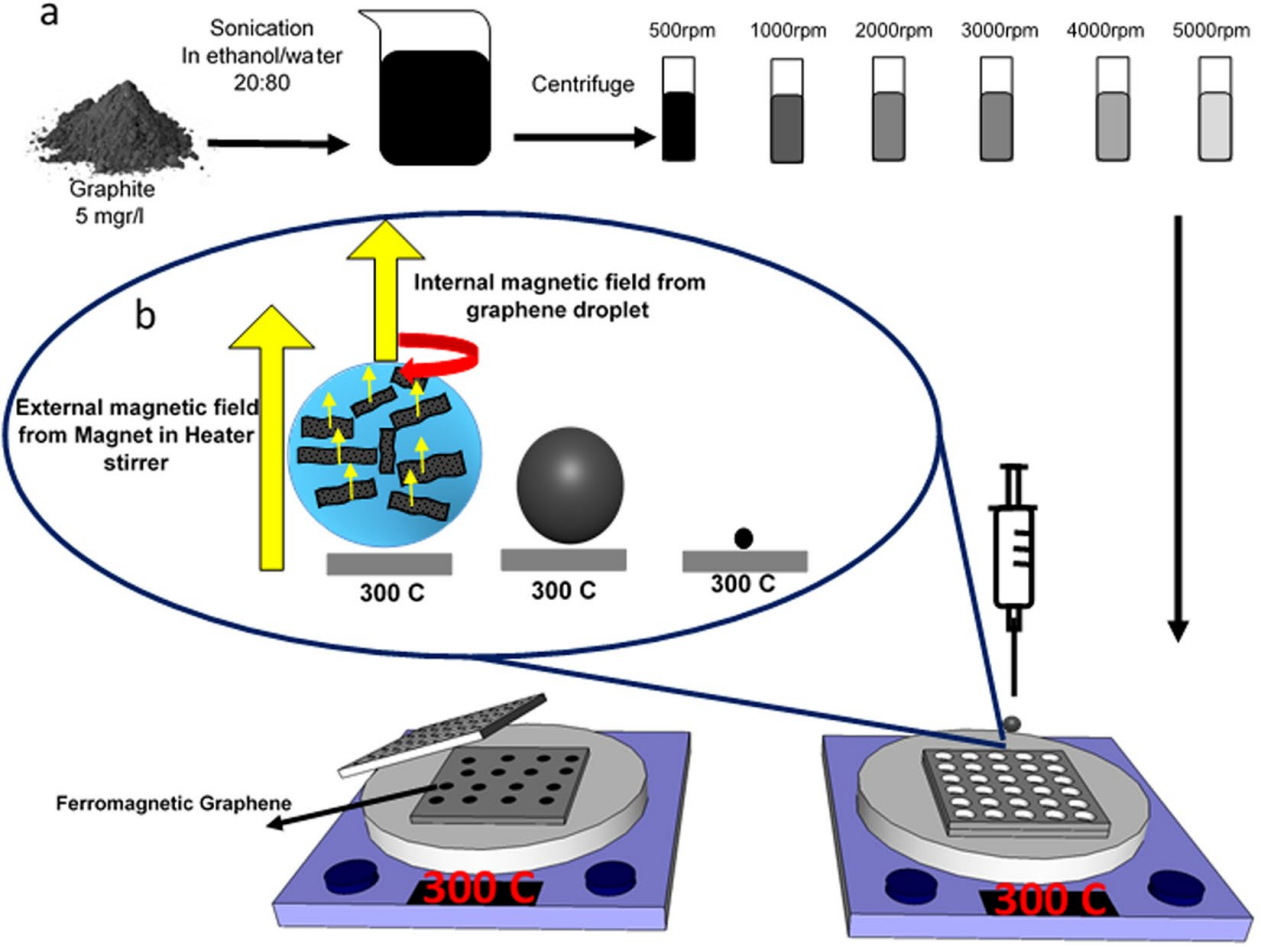
Scheme of Leidenfrost effect-based method. (**a**) The liquid-phase exfoliation method for graphene dispersion. Graphite is sonicated in ethanol/water solution and is centrifuged at various speed (500–5000 rpm). In the following, the suspension is injected to the aluminum holes. This set up is provided to apply the Leidenfrost effect to graphene droplets. (**b**) Evaporating the Leidenfrost droplets in the external and internal magnetic field. The internal magnetic field is created by circulation of the graphene inside the droplets under the external magnetic fields.

To apply the Leidenfrost effect to the graphene droplets, two aluminum plates are placed on the heater surface (300 °C). In order to control the bouncing of the droplets, the upper plate is drilled. The volume of each hole is about 0.05 ml. As shown in Fig. 1, the perforated aluminum plate is placed on the bottom plate, which acts as a substrate, consequently is used for better collecting the FGPs. The graphene suspension is dropped in the holes by syringe. The droplets float and slowly evaporate; so that after about 1 min, the ethanol and water evaporate, and the ferromagnetic graphene particles remain on the substrate. Subsequently, the particles are collected for further characterization. The yield of FGP is measured in the range of 0.33–0.02% for various concentrations.

## Results and discussion

In recent findings, mechanical control over the electronic structure of graphene is known as “strain engineering”, which affects the magnetic properties of graphene. For more explanation, the pseudo-magnetic field of highly strained nanobubbles of graphene is reported by the N. Levy et.al in 2010. In this study, in addition to strain, other parameters such as temperature and the magnetic fields are applied to graphene flakes by the Leidenfrost effect-based method. Temperature and magnetic field, like strain, can change the electronic structure of graphene flakes and cause magnetic properties in these flakes.

### Electronic structure of FGPs

**Figure 2:**
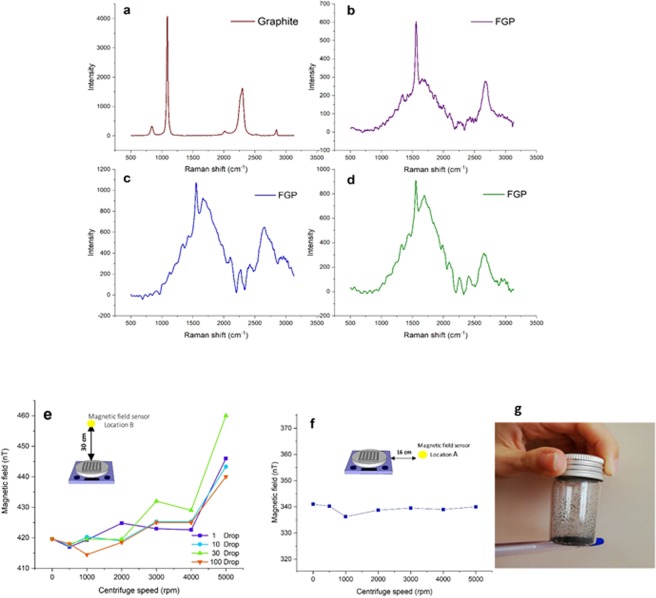
The Raman spectra and the magnetic field sensor data. (**a**) Raman scattering of graphite. (**b**–**d**), Raman scattering of three different points of FGPs at 3000 rpm (**e**), the magnetic field sensor is placed on 30 cm above the heater stirrer (inset). The increasing of the magnetic field by increasing the centrifuge speed. The external magnetic field related to the heater stirrer magnet is about 420 (±6% uncertainty) and the internal magnetic fields related to droplets are about 1–5 nT. This diagram show the internal magnetic fields of different number of droplets (1, 20, 30, 100 droplet). (**f**) The magnetic field sensor is placed on 16 cm near to heater stirrer (inset). No magnetic field is detected so the fluctuation of the magnetic field is negligible. (**g**) Static charge response of FGPs to chargeable pen, polishing by cloth.

Raman spectra of single and multilayer graphene indicate useful and prominent features based on the phonon and electronic properties of them. In addition, the changes of lattice vibration and electronic structure in the presence of external perturbations such as magnetic field, strain, and temperature can be determined through the Raman spectroscopy. The three main scattering modes for graphene systems located at ~1340, 1574, and 2707 called D, G (normal first order Raman scattering process) and 2D, respectively. The Raman spectrum of pristine graphite is shown in Fig. 2a which has three main peaks^61^.

In Fig. 2(b–d), the peculiar Raman spectra are represented for FGPs at 3000 rpm. The parameters involved in transition and scattering processes inside FGPs is too much that investigating these processes is not possible. Up to now, the spectra of graphene have separately been investigated under different external perturbation in the size of nanoscale and ideal form^61^. Indeed, the frequency of Raman peaks related to temperature^62^, is raised by isotropic compression and reduced by isotropic tension^63^. Also, anisotropic stress gives complex features to these peaks (splitting the G mode). In addition, Landau levels can be generated due to the application of an external magnetic field (and in some cases by strain)^64–71^, which transition between these levels lead to magneto excitation create^72^. Resonance, magneto phonon resonances, occurs when the energy between the levels of the Landau and the lattice vibrations of graphene is equal. Other parameters such as number of layers and stacking form can influence Raman spectra. Therefore, we confirm just the change of electronic structure in the graphene by Raman spectra because the graphene flakes inside the droplets feel the non-uniformed perturbation applied in any direction^61^. The starting point of these changes is observed when the Leidenfrost droplets are placed on the holes. The magnetic field sensor measures the weak magnetic fields for droplets as an internal magnetic field based on the circulation graphene flakes inside the droplets (Fig. 2c). In fact, the graphene flakes act as a charged particle due to change of electron distribution and also produce magnetic field by their moving (Fig. 2f). Also, static charge is observed in FGPs (Fig. 2g and Video 1). Generally, these changes do not relate to functionalization which is confirmed by IR spectrum (Supplementary Fig. S3).

#### Magnetic fields and strain

In recent studies, responding of graphene to external factors have been reported such as an alignment of graphene sheets under the external magnetic field^73^ and generating the pseudo-magnetic field by strain engineering^66,68,71^. As mentioned above, graphene properties are affected by graphene forms (suspended and supported). Here, under the magnetic field, the floated graphene flakes circulate inside the droplet and aggregate during the evaporation. As explained before, the dynamic inside the Leidenfrost droplets is not clear, also, the investigation of the floated graphene inside the droplet is not possible. Therefore, due to these limitations, the probable magnetization process is proposed and interpreted based on the magnetic field measurements. Magnetic field measurement with this procedure detects two sources of magnetic fields. One of them is external, about 420 nT generated by the magnet inside the heater-stirrer, and other is an internal magnetic field in the region of 1–5 nT generated by the circulation of the polarized graphene flakes inside the droplets. Generation of the internal magnetic field contains two steps: (1) the polarization of graphene under the external perturbation when the graphene suspension is injected in the aluminum holes, (2) generation of the internal magnetic field through the circulation of polarized graphene, based on Faraday law.

In addition, the polarizability of graphene sheets depends on the number of layers and influence the internal magnetic field. To elaborate more, as shown in Fig. 2e, with an increase in the centrifuge speed (decreasing the number of layers), the internal magnetic field is risen, because in the multilayer graphene, the inner electrons involve the Van der Waals interlayer interactions, thus they are less affected by the external magnetic field. Also, the inner electrons of graphene have less potential for polarization. Moreover, for different number of droplets, the total magnetic field inside droplets as a function of centrifuge speed have been plotted in Fig. 2e, which allows us to establish a simple interpretation about the rotational features of the Leidenfrost droplets. Indeed, a negligible increase in this field relates to the various rotate direction of droplets which weakens magnetic field of each other.

Due to high temperature of the heater, measurement of magnetic field faces limitations; therefore, the sensor is located at 30 cm above the heater, as seen in the inset of Fig. 2e. In general, measuring the magnetic field is directly related to the distance. For comparison, the measured magnetic field is about 450 nT at 30 cm above the heater and is about 1 μT on the surface of heater (when the heater is off). Therefore, it is predicted that the internal magnetic field is also more than 1–5 nT. When the sensor is positioned at 16 cm near the heater, no magnetic field changes are observed, hence the environmental fluctuations are almost zero (Fig. 2f).

**Figure 3:**
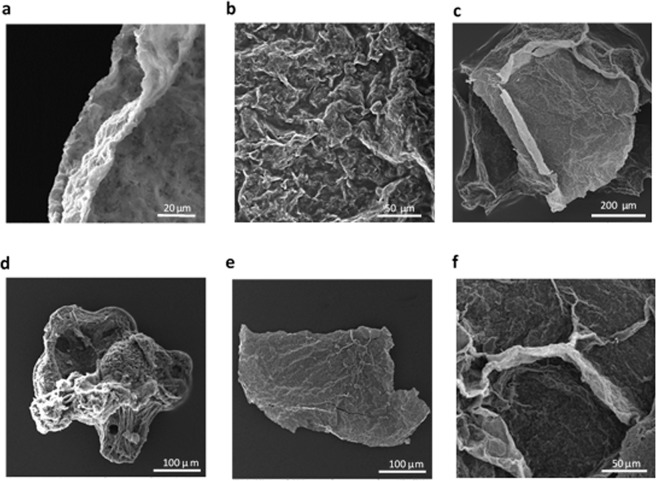
FESEM images of FGPs. (**a**–**f**) show different shapes of FGPs and the wrinkles which is created under the strain inside the Leidenfrost droplets for 500 to 5000 rpm respectively.

Also, high and non-uniform strain is applied on graphene flakes, which creates different shapes and wrinkles on FGPs. Figure 3 shows a typical field emission scanning electron microscopy (FESEM) image from our samples. The layers are closely packed to each other similarly to graphite. The values of surface area for graphite and FGPs (with 500 rpm) which have been measured by using Brumauer-Emmett-Teller (BET) are 7.3678 m^2^/gr and 6.2320 m^2^/g, respectively that are close to each other.

### Ferromagnetic graphene particles characterization

**Figure 4:**
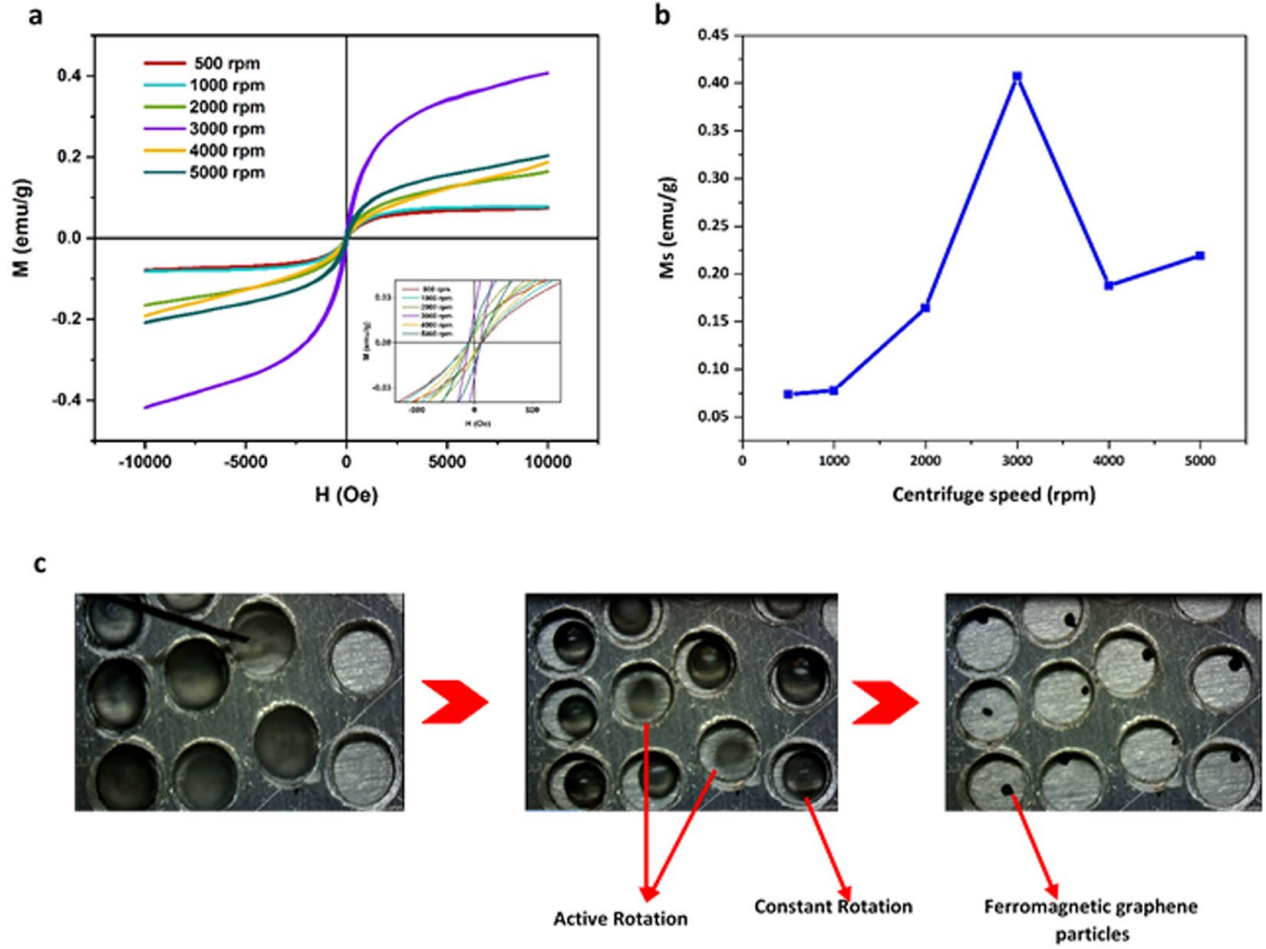
The vibrating sample magnetometer (VSM) for all FGPs and rotational style. (**a**) The hysteresis loop for the FGPs at various speed (500–5000). Inset is zoom in −100 to 100 Oe, the data was summarized in Table 1. (**b**) Show the magnetization saturation (Ms) vs the centrifuge speed. (**c**) Top view of the procedure for 2000 rpm. and the rotational style, constant rotation (CR) and the active rotation (AR), two droplet rotate as active rotation and others rotate as constant rotation.

The vibrating sample magnetometer (VSM) was performed for all samples. The (M-H) curve for graphite and graphene shows weak ferromagnetic properties (Supplementary Fig. S4). As shown in Fig. 4a, the magnetization hysteresis loops are measured at room temperature in the field range of −10 kOe < *H* < +10 kOe. Table 1 shows saturation magnetization (Ms), coercive field (Hc), and remanence magnetization (Mr) for all samples. The higher magnetization relates to sample with 3000 rpm which has 0.4 emu/g as a saturation magnetization. Furthermore, the measurements show weak ferromagnetic behavior of graphite (Supplementary Fig. S4a) usually related to the defects and negligible impurities^21–24^ of graphite investigated by Raman and ICP spectroscopy (Supplementary Table S1), which is a consequence of the fact that the magnetic behavior of FGPs is not related to pristine graphite, because all the samples were prepared from a same source. As summarized in Table 1, the coercive field and remanence magnetization do not follow certain pattern; for example, the maximum of Mr belongs to 3000 rpm.

**Table 1:** Saturation magnetization (Ms), coercive field (Hc) and remnant magnetization (Mr) for all samples.

Centrifuge speed (rpm)	Ms (emu/g) H = 10,000 Oe	Mr (emu/g)	H_c_ (Oe)
500	0.07375	0.00595	~60
1000	0.07775	0.00601	~60
2000	0.1644	0.00756	~40
3000	0.40725	0.01736	~40
4000	0.18786	0.00416	~40
5000	0.21924	0.01012	~70

As shown in Fig. 4b, the saturation magnetization is raised by increasing the centrifuge speed until 3000 rpm and after that reduced. In general, the magnitude of the FGPs magnetization depends on three parameters including the number of layers, internal magnetic field, and the rotational style. As mentioned above, with increasing speed of the centrifuge, the number of layer is reduced and consequently, the internal magnetic field is raised. The role of the internal magnetic field in increasing of the saturation magnetization is low, because contribution of the internal magnetic field to the external magnetic field is negligible. As noted above, with an increase in the centrifuge speed, the number of graphene layers is reduced. Fewer electrons involve with the interlayer Van der Waals interaction that cause electronic structure of graphene, which has fewer layers, to change easier. Therefore, we expect a risen in the Ms by increasing the centrifuge speed. A decrease in magnetism at 4000 and 5000 rpm depends on the rotational style of the droplets. Two rotational style are observed in this procedure, including the constant rotation (CR) and the active rotation (AR), as seen in Fig. 4d and Video 2. At each rate, 100 drops were investigated, and the results were summarized in Table 2. Although the reason for the difference of the rotational behavior of droplets at different speeds is unclear, the enhancement of percent of the droplets with active rotation style cause magnetism to be reduced in comparison with constant rotation. In 4000 and 5000 rpm, most of the droplets rotate in active style. Lifetime of droplets is decreased by increasing the centrifuge speed related to quickly evaporation of active rotation style (Table 2).

**Table 2:** Lifetime and the rotational style, Constant Rotation (CR) and Active Rotation (AR).

Centrifuge speed (rpm)	Life time (s)	CR	AR
500	110.45	78%	22%
1000	113.34	74%	26%
2000	107.09375	59%	41%
3000	106.83	50%	50%
4000	103.97	47%	53%
5000	99.38	34%	66%

## Conclusions

We have introduced a new method to produce ferromagnetic graphene particles at macroscopic scale. In this method, electronic structure of exfoliated graphene is changed using magnetic field, strain, and temperature. The magnetization of FGPs is measured ~0.4 emu/g at room temperature, which is almost higher than the previous reports. This method is economical, simple, and fast, which obtains the products without residual chemicals. Also, environmental advantage due to usages of green materials can be considered. Beside of measuring magnetization, the electronic system and morphologies of FGPs are investigated. In addition, magnetic field of Leidenfrost droplets is revealed by detection sensor. Two different rotational style and different direction of rotation are observed, which their percent in droplets affect their magnetization properties.

## Materials and Characterization

The graphite and ethanol (99.9%) are purchased from Merck Company. Larger flakes are collected by centrifuge (Hettich EBA20). Set up contains heater stirrer (Heidolph, MR Hei-standard) and two aluminum plates (thickness 2.45 mm). Almost one hundred hole (4.76 mm, 0.05 ml) is drilled in the top plate. The impurities of graphite are measured by ICP spectrometry (ICPS-7000 SHIMADZU). Adjusting surface tension of ethanol solution is performed using Tensiometer (sigma 700 ring mode). Magnetization of FGPs is measured by vibrating sample magnetometer (Kavir IRAN). Magnetic field sensor (Narba, NBM550, German) is used to detect magnetic fields. Raman spectra are obtained by Raman spectroscopy (HORIBA, 532 nm). The morphologies are investigated by FESEM (TESCAN). UV-Vis (mini 1240) and FTIR (8400S) spectrophotometer analyzes are performed by SHIMADZU instruments. The specific surface area is determined by Micromeritics instrument.

## Supplementary information

Supplementary information.
