# Supplementary material for: Manipulating electronic structure of graphene for producing ferromagnetic graphene particles by Leidenfrost effect-based method
Source: Sci Rep. 2020 Apr 23;10:6874. doi: 10.1038/s41598-020-63478-7 (PMC7181710; doi:10.1038/s41598-020-63478-7)
Supplement: Supplementary file 1 — Supplementary information. [file 41598_2020_63478_MOESM1_ESM.docx]

Supplementary Information for

**Manipulating electronic structure of graphene for producing ferromagnetic graphene particles by Leidenfrost effect-based method**

*

Mahsa Alimohammadian ^a^, Beheshteh Sohrabi *^[[1]](#footnote-1), a^

*^a^ Department of Chemistry, Surface Chemistry Research Laboratory, Iran University of Science and Technology, 16846-13114, Tehran, Iran*


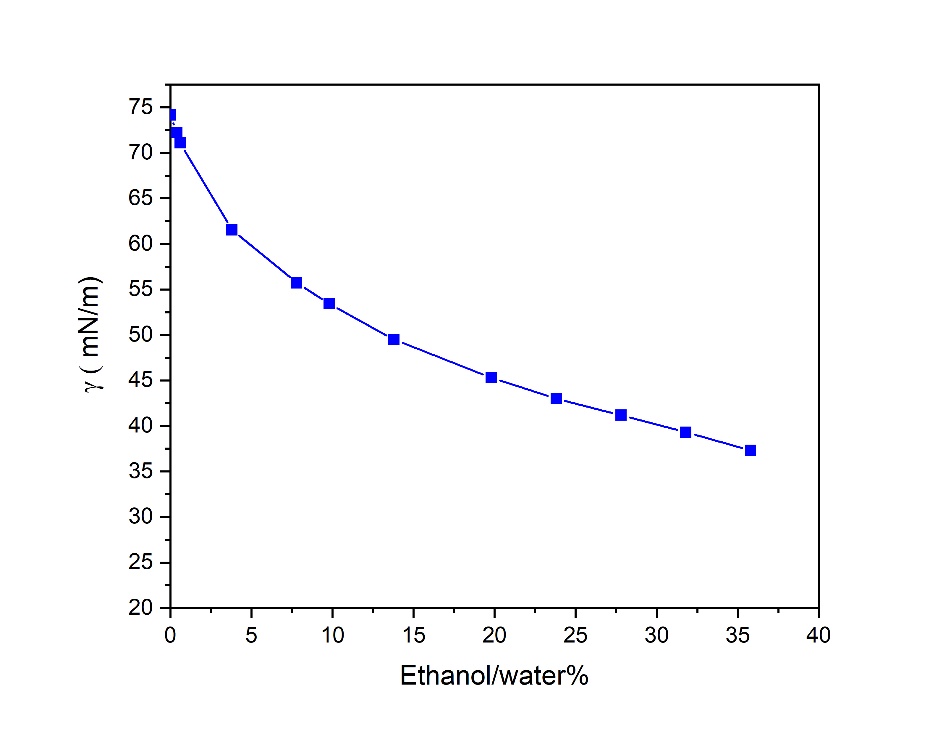


**Supplementary Figure S1| Adjusting surface tension for ethanol solution.** For obtaining the surface tension of ~45 ± 0.1 mN/m, the ratio of ethanol/water solution is estimated ~20:80.


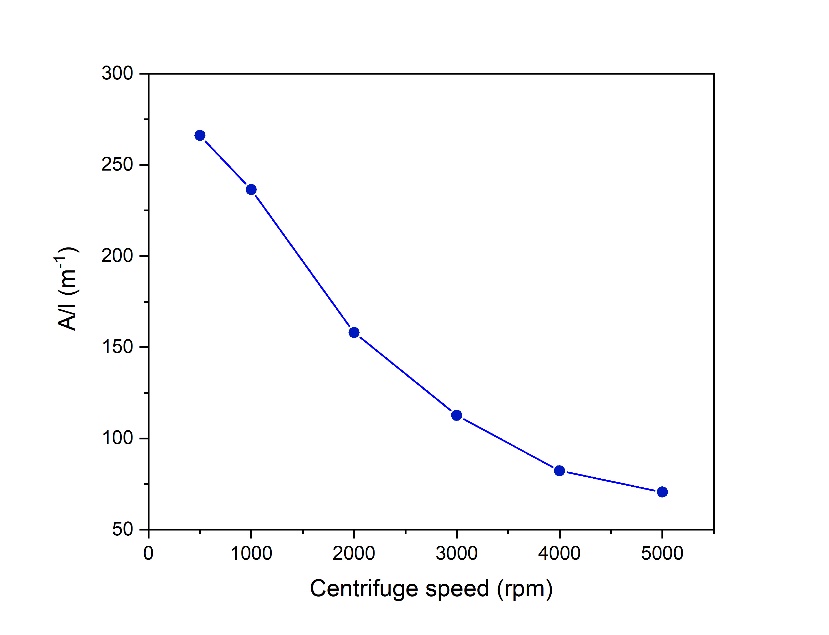


**Supplementary Figure S2 | The yield of graphene in various centrifuge speed**. The UV-Vis absorption at 660 nm is recorded for each samples and is divided by length cell (± 0.04). According to the Beer-Lambert equation and 3182 L/gr.m (Ref.55 ) as a coefficient, the concentration of graphene is calculated.


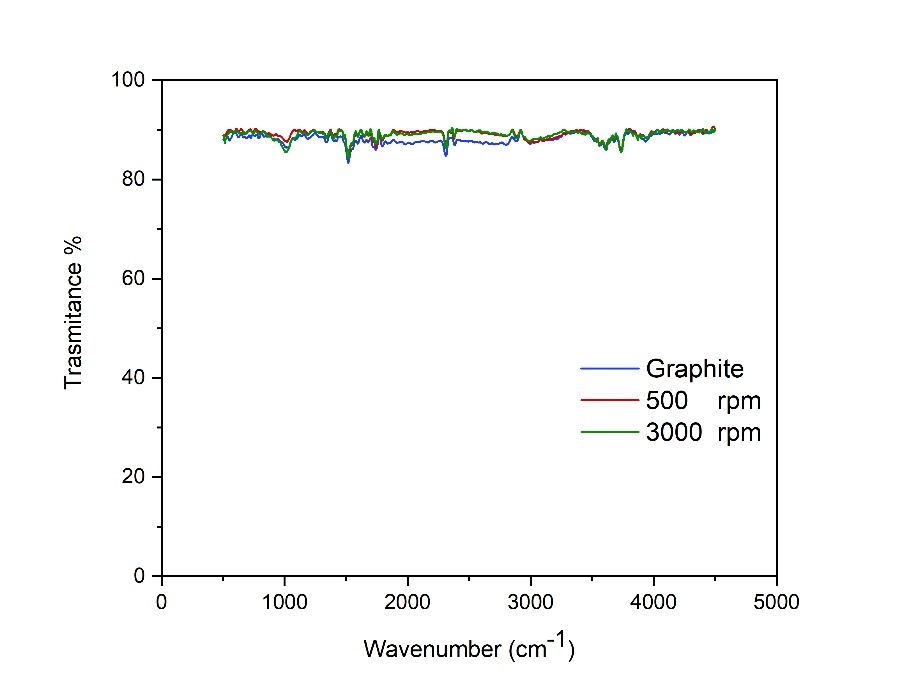


**Supplementary Figure S3 | FTIR spectra for graphite and FGPs at 500 and 3000 rpm.** No absorption is observed so functionalization is not occurred.


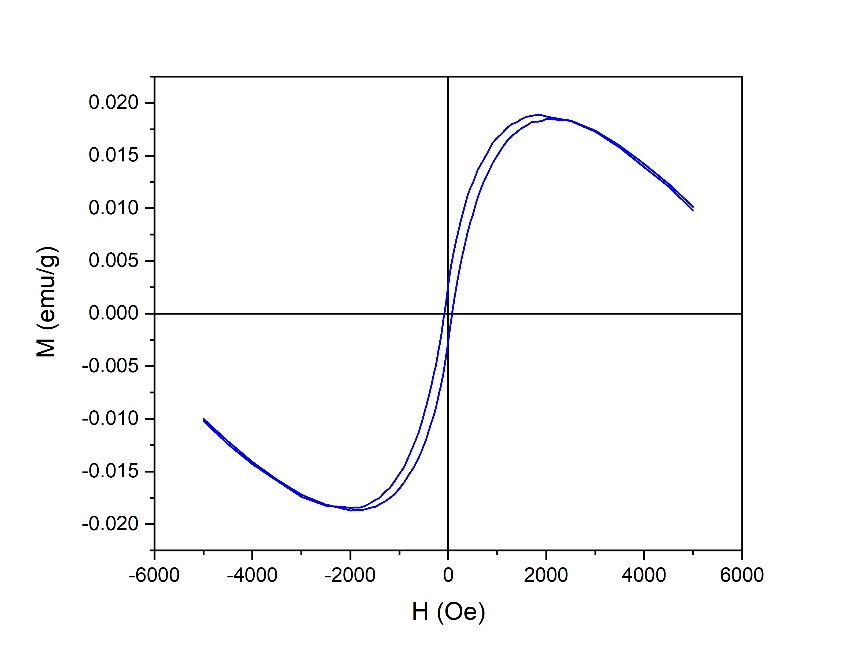

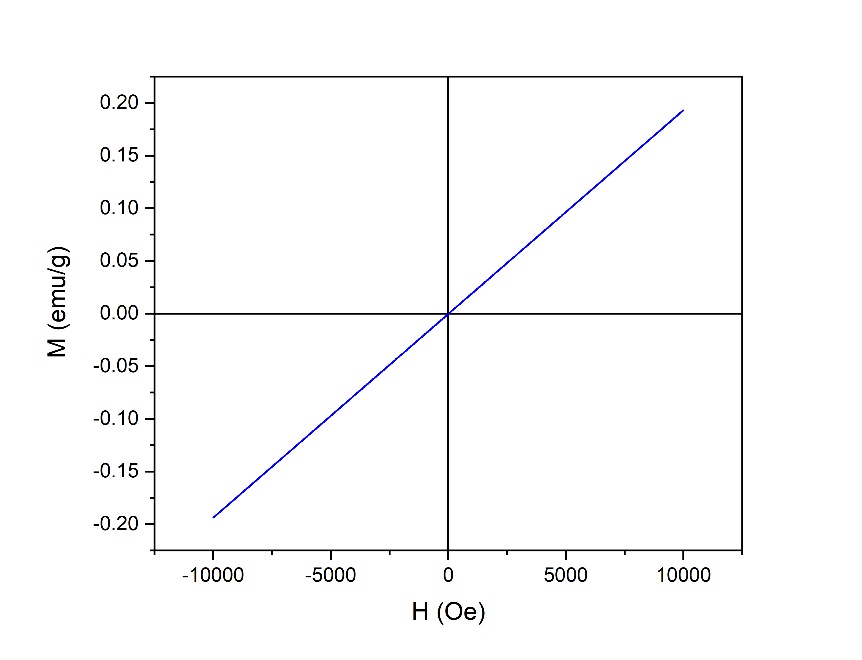


a

b

**Supplementary Figure S4 | Magnetization of graphite and graphene**. a The M-H curve of graphite. Show weak ferromagnetic properties. b. show paramagnetic diagram for exfoliated graphene.

**Supplementary Figure S4 | Magnetization of graphite and graphene**. a The M-H curve of graphite. Show weak ferromagnetic properties. b show paramagnetic diagram for exfoliated graphene.

| **Supplementary Table S1 \| Impurities of pristine graphite.** | | | | |
| --- | --- | --- | --- | --- |
| element | Fe | Ni | Co | Mn |
| Concentration | >1ppm | >0.1 ppm | <0.1 ppm | <0.1 ppm |

1. * Corresponding author: [Sohrabi_b@iust.ac.ir](mailto:Sohrabi_b@iust.ac.ir), [Sohrabi_b@yahoo.com](mailto:Sohrabi_b@yahoo.com) [↑](#footnote-ref-1)
